# Assessing the knowledge, attitudes and practices of physicians on perioperative antibiotic prophylaxis in pediatric surgery in China: a descriptive study

**DOI:** 10.3389/fped.2026.1884110

**Published:** 2026-07-09

**Authors:** Yao Sun, Chuan Sun, Feng Chen, Jin Xu, Lihua Yuan

**Affiliations:** Department of Pharmacy, Children’s Hospital of Nanjing Medical University, Nanjing, China

**Keywords:** antimicrobial stewardship, cluster analysis, knowledge, attitude, practice, perioperative antimicrobial prophylaxis, surgical site infection

## Abstract

**Background:**

Surgical site infection (SSI) prevention is a critical component of pediatric surgical safety, yet the irrational prolongation of perioperative antimicrobial prophylaxis (PAP) remains a global driver of antimicrobial resistance (AMR). While guidelines explicitly recommend discontinuing prophylaxis within 24 h for clean incisions, compliance varies significantly. This study aimed to evaluate the knowledge, attitudes, and practices (KAP) of pediatric surgeons regarding PAP and to identify the determinants of non-compliant behaviors using a novel clustering approach.

**Methods:**

A multicenter, cross-sectional study was conducted from July to August 2025 across Children's hospitals in Jiangsu Province, China. A total of 143 surgeons from different surgical departments (e.g., General Surgery, Orthopedics, Neurosurgery, Cardiothoracic Surgery) were surveyed using a validated questionnaire based on WHO guidelines. Data were analyzed using Spearman's rank correlation to assess knowledge-practice associations and K-Means clustering to identify distinct behavioral patterns among clinical departments.

**Results:**

The study achieved a valid response rate of 95.3%. While surgeons demonstrated high cognitive accuracy regarding drug selection (>84.0%) and administration timing (95.1%), only 74.8% acknowledged the standard of discontinuing prophylaxis within 24 h for Class I incisions. A significant positive correlation was found between knowledge and practice scores (*r* = 0.579, *P* < 0.001). However, cluster analysis revealed a “Knowledge-Practice Disassociation” pattern in the Oncology, Burns, and SICU departments.

**Conclusion:**

A high level of cognitive reserve cannot ensure consistent implementation of standard practices. In high-risk departments, clinicians' focus on SSI rather than AMR may trigger defensive medicine, which is likely to impair guideline adherence. This association requires further verification in targeted investigations.

## Introduction

Perioperative prophylactic use of antimicrobial agents is a core strategy for reducing the incidence of surgical site infections (SSIs) and ensuring the safety of surgical patients, and the rational selection and administration of antimicrobials constitute an important means of effectively preventing postoperative infections (1, 2). Conversely, inappropriate prophylactic use of antimicrobial agents can readily induce the emergence of drug-resistant strains, which not only increases the difficulty of treating clinical infections and raises healthcare costs, but also significantly delays the postoperative recovery process in pediatric patients (3). As the principal agents responsible for antimicrobial use in clinical practice, surgeons' level of knowledge regarding perioperative antimicrobial application and their clinical decision-making capacity directly determine the rationality of antimicrobial use (4).

With the gradual strengthening of hospital infection-prevention awareness among surgeons, the appropriateness of prophylactic antimicrobial use in pediatric class I surgical incisions has improved substantially. However, determining the optimal timing to discontinue antimicrobial prophylaxis remains a major clinical challenge, especially in pediatric patients. Their immature physiological function leads to unique pharmacokinetic profiles and varying drug tolerance. Meanwhile, factors including severe surgical trauma and surgical implant placement greatly elevate the risk of perioperative infection, which often prompts clinicians to prolong antimicrobial use. Consequently, establishing an evidence-based strategy for the timely discontinuation of perioperative prophylactic antimicrobial therapy in pediatric class I incision surgery remains a pressing and unresolved issue in clinical practice (5).

This study employed a targeted questionnaire survey covering pediatric hospitals across all prefecture-level cities in Jiangsu Province to systematically assess the current status of knowledge, attitudes, and practices (KAP) regarding perioperative antibiotic prophylaxis among pediatric surgeons. The findings aim to provide an evidence-based foundation for standardizing the refined management of prophylaxis in pediatric Class I (clean) incisions and enhancing the overall rationality of clinical antimicrobial usage.

## Methods

### Study design and participants

This multicenter, cross-sectional study was conducted between July and August 2025 across pediatric specialty hospitals in all prefecture-level cities in Jiangsu Province, China. According to the official data from hospital websites reports: The total number of full-time pediatric surgeons in tertiary children's hospitals in Jiangsu Province is approximately 260. Eligible participants comprised practicing surgeons with prescribing authority across departments of general surgery, neurosurgery, cardiothoracic surgery, and orthopedics.

### Sampling and sample size

A combination of purposive and convenience sampling methods was used to recruit participants. The sample size was calculated using the formula for estimating a single proportion:

$$n=\frac{Z^{2}p(1-p)}{e^{2}}$$

A 95% confidence level (*Z* = 1.96), an expected proportion *p* = 0.5 (conservative estimate to maximize sample size), and an allowable error *e* = 0.05 were used, yielding an initial sample size of 385.

Since the target population of pediatric surgeons in tertiary children's hospitals in Jiangsu Province is small (260), a finite population correction (FPC) was applied:

$$n_{\mathrm{corrected}}=\frac{n_{0}}{1+\frac{n_{0}-1}{N}}$$

Using the population size *N* = 260, the corrected minimum sample size was 156. After adjusting for an estimated 5% non-response rate, the final target sample size was set to 165. The 150 valid questionnaires collected in this study are close to the corrected theoretical value and represent more than 50% of the total target population, ensuring good representativeness.

### Questionnaire design and pilot study

The questionnaire was developed using the Knowledge-Attitudes-Practices (KAP) framework, with items derived from authoritative clinical guidelines, including the WHO Global Guidelines for the Prevention of Surgical Site Infection (2016) (6, 7). Prior to formal data collection, a pilot study involving 10 surgeons was conducted to refine and streamline the items, resulting in a finalized 21-item questionnaire. The formal questionnaire consisted of four sections: (1) demographic and professional characteristics; (2) knowledge of perioperative antibiotic prophylaxis (5 items), focusing on indications, drug selection, administration timing, and duration of therapy for Class I incisions; (3) attitudes toward prophylaxis (9 items), assessed using a 5-point Likert scale (ranging from 1 = strongly agree to 5 = strongly disagree); and (4) clinical practices (8 items), examining drug selection criteria, decision-making regarding duration of therapy, and consultation patterns. We randomly recruited eligible surgeons from cooperating hospitals and distributed electronic questionnaires via online platforms. The survey was fully anonymous and confidential, with no personal identifiable information collected.

### Statistical analysis

Data were managed and cleaned using Microsoft Excel, while statistical analyses and visualizations were performed using Python and SPSS version 26.0.

(1) Descriptive Statistics: Continuous variables following a normal distribution are presented as mean ± standard deviation (SD), while categorical data are expressed as frequencies (*n*) and percentages (%).
(2) Individual-Level Knowledge-Practice Association: To investigate the impact of cognitive levels on clinical behavior, a quantitative KAP scoring model was constructed. Spearman rank correlation coefficient was used to assess the relationship between knowledge and practice scores, and the Mann–Whitney *U*-test was applied to compare practice scores between high-knowledge and low-knowledge subgroups.
(3) Department-Level Cluster Analysis: To identify behavioral patterns across clinical specialties, data were aggregated by department to calculate mean knowledge and practice compliance rates. The Wilcoxon signed-rank test was used to evaluate paired differences between knowledge and practice rates within departments, Pearson correlation analysis was conducted to evaluate the linear association between departmental knowledge rates and practice adherence rates. Departments were further categorized into distinct behavioral clusters using the K-means clustering algorithm based on their respective knowledge and practice rates.
(4) All statistical tests were two-sided, with a *P*-value < 0.05 considered statistically significant.

## Results

### Demographic and professional characteristics

**Figure 1:**
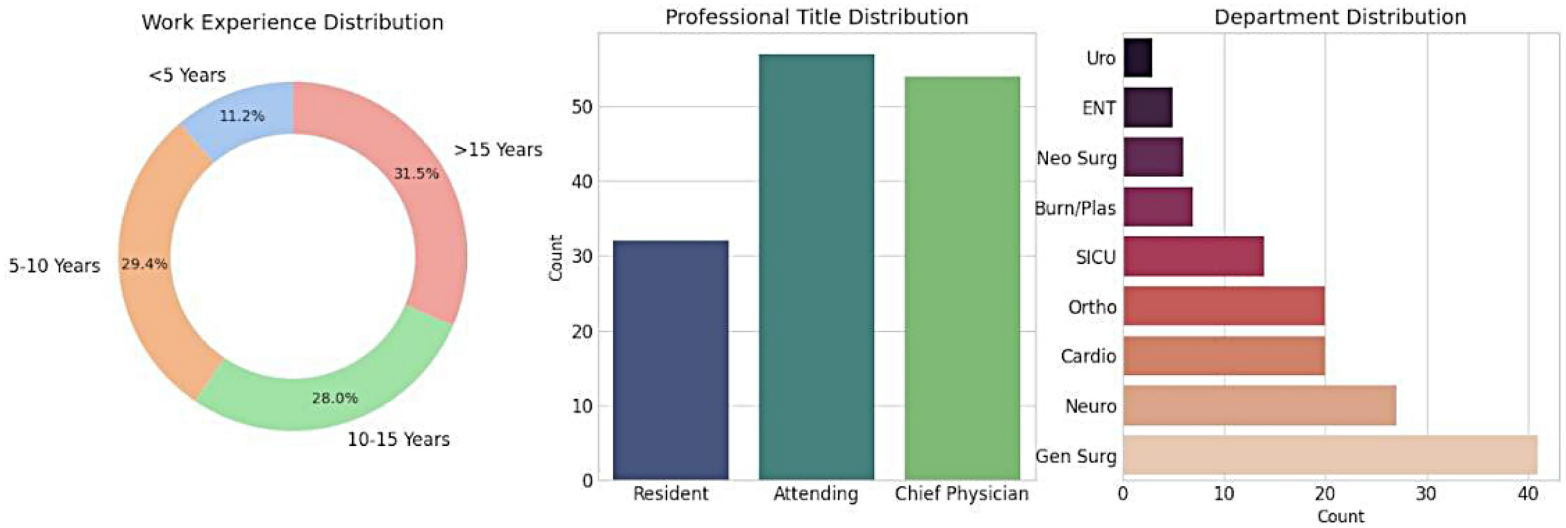
Demographic and professional characteristics.

Among the 143 surgeons included in the analysis, 131 (91.6%) were male; regarding professional rank, attending and senior physicians collectively accounted for 77.6%, and 31.5% had more than 15 years of clinical experience. In terms of training, 81.8% of the participants had received specialized training on the rational use of antimicrobials within the preceding two years (Figure 1).

### Knowledge of perioperative antimicrobial prophylaxis

Knowledge accuracy regarding the optimal timing of prophylactic antimicrobial administration (0.5–1 h preoperatively) attained 95.1%, and the accuracy concerning drug selection criteria reached 84.0% or higher. However, substantial gaps were observed in knowledge of appropriate discontinuation timing, as only 74.8% of physicians correctly recognized that prophylaxis for pediatric Class I incisions should be terminated within 24 h postoperatively, with non-concordance primarily concentrated in departments involving prolonged surgical duration and substantial operative trauma (Tables 1, 2).

**Table 1:** Knowledge items and results concerning perioperative antimicrobial use and resistance.

Statement items	Agree	Disagree	Not sure
Prophylactic antimicrobials are indicated for pediatric patients undergoing clean (Class I) surgical procedures.	69 (48.3%)	71 (49.7%)	3 (2.1%)
Routine antimicrobial prophylaxis is necessary for all categories of surgical incisions in pediatric perioperative care.	9 (6.3%)	123 (86.0%)	11 (7.7%)
The optimal timing for perioperative antimicrobial administration is 0.5 to 1 h prior to the surgical incision.	136 (95.1%)	6 (4.2%)	1 (0.7%)
For pediatric Class I (clean) incisions, cefazolin or cefuroxime are the primary agents of choice for prophylaxis.	134 (93.7%)	0 (0.0%)	9 (6.3%)
The recommended duration for prophylactic antimicrobial use in pediatric Class I (clean) procedures is within 24 h postoperatively.	107 (74.8%)	23 (16.1%)	13(9.1%)

**Table 2:** Knowledge and practice of participants regarding perioperative antimicrobial prophylaxis.

Department	Count (*n*)	Percentage (%)	Knowledge: Discontinuation within 24 h (*n*)	Proportion of Department Respondents (%)	Practice: Discontinuation within 24 h (*n*)	Proportion of Department Respondents (%)
General Surgery	41	28.7	35	85.4	33	80.5
Neurosurgery	27	18.9	21	77.8	20	74.1
Orthopedics	20	14.0	18	90.0	16	80.0
Cardiothoracic Surgery	20	14.0	10	50.0	7	35.0
Surgical ICU	11	7.70	11	100.0	5	45.5
Burns and Plastic Surgery	7	4.9	7	100.0	2	28.6
Otolaryngology (ENT)	5	3.5	5	100.0	5	100.0
Oncology	5	3.5	5	100.0	1	20.0
Neonatal Surgery	4	2.8	3	75.0	3	75.0
Urology	3	2.1	2	66.7	1	33.3

### Attitudes toward perioperative antimicrobial prophylaxis

Attitudinal inclinations toward pediatric perioperative antimicrobial prophylaxis were assessed via nine items (Table 3); the results indicated a high level of consensus regarding the necessity of avoiding antimicrobial overuse and an acute awareness of the associated risks, with the specific distribution as follows: 101 (70.6%) participants identified Gram-positive cocci as the predominant pathogens in perioperative infections; 122 (85.3%) believed that perioperative antimicrobial usage should undergo periodic evaluation; 84 (58.7%) recognized that prolonged antimicrobial therapy increases the risk of bacterial resistance; 140 (97.9%) understood that excessive duration of perioperative antimicrobial prophylaxis may induce the selection of resistant strains; 133 (93.0%) considered prolonged prophylaxis likely to increase the incidence of adverse drug reactions; 130 (90.9%) agreed that such practices disrupt the homeostasis of the patient's intestinal microbiota; 112 (78.3%) realized that excessive medication escalates healthcare expenditures. Notably, neutral responses to these attitude questions were mainly reported by clinicians working in cardiothoracic surgery. This raises concerns that merely 36.4% (*n* = 52) of physicians agreed that keeping up with the latest domestic and international guidelines on pediatric antimicrobial use is essential for rational prophylactic medication.

**Table 3:** Attitudes toward perioperative antimicrobial use and antimicrobial resistance.

Attitude statements	Strongly agree	Agree	Neutral	Disagree	Strongly disagree
Gram-positive cocci should be the primary focus during the perioperative period, while coverage for Gram-negative bacilli should also be considered in cases involving contaminated incisions.	101 (70.6%)	40 (28.0%)	2 (6.3%)	0 (0.0%)	0 (0.0%)
The necessity of antimicrobial use should be assessed periodically during the postoperative period.	122 (85.3%)	18 (12.6%)	3 (3.2%)	0 (0.0%)	0 (0.0%)
Long-term use of antimicrobials increases the risk of developing bacterial resistance.	84 (58.7%)	45 (31.8%)	4 (2.8%)	10 (7.0%)	0 (0.0%)
Excessive use of antimicrobials may increase the risk of inducing the emergence of resistant strains.	140 (97.9%)	3 (3.2%)	0 (0.0%)	0 (0.0%)	0 (0.0%)
Excessive use of antimicrobials may increase the risk of adverse drug reactions, such as hepatic or renal impairment and allergic reactions.	133 (93.0%)	3 (5.6%)	2 (6.3%)	0 (0.0%)	0 (0.0%)
Overuse of antimicrobials disrupts the homeostatic balance of the intestinal microbiota.	130 (90.9%)	10 (7.0%)	3 (3.2%)	0 (0.0%)	0 (0.0%)
Excessive antimicrobial use leads to an escalation in healthcare expenditures.	112 (78.3%)	20 (14.0%)	11 (7.7%)	0 (0.0%)	0 (0.0%)
Pharmacists play a pivotal role in the implementation of antimicrobial stewardship programs.	138 (96.5%)	5 (3.5%)	0 (0.0%)	0 (0.0%)	0 (0.0%)
Healthcare professionals should remain abreast of the latest domestic and international clinical guidelines for pediatric antimicrobial use.	52 (36.4%)	74(51.5%)	17(11.9%)	0(0.0%)	0(0.0%)

### Clinical practice in perioperative antimicrobial prophylaxis

This study assessed the clinical practice of pediatric perioperative antimicrobial prophylaxis through seven items (Table 4), with results summarized as follows. Regarding therapeutic decision-making, 82.5% of the participants formulated antimicrobial regimens by synthesizing guideline recommendations, hospital drug formularies, and the distribution of common pathogens at the surgical site. For Class I incisions, 67.8% of clinicians prioritized first- or second-generation cephalosporins as the primary agents for prophylaxis. Nearly half of the respondents optimized individualized treatment decisions by further considering patient hepatic and renal function, drug metabolic profiles, and current antimicrobial resistance patterns. Furthermore, 94.4% of physicians performed microbiological cultures prior to prescribing antimicrobials when perioperative infection symptoms occurred. In cases of infection involving resistant pathogens, 90.9% of clinicians initiated multidisciplinary consultation mechanisms.

**Table 4:** Clinical practice in perioperative antimicrobial prophylaxis.

Antimicrobial selection criteria	National or international clinical guidelines	Hospital antimicrobial formulary restrictions	Targeted coverage of common pathogens at the surgical site	Hepatic and renal function and drug metabolic profiles	Antimicrobial resistance surveillance data
References for antimicrobial selection when issuing perioperative orders (multiple choices)	118 (82.5%)	101 (70.6%)	105 (73.4%)	62 (43.4%)	59 (41.3%)
Antimicrobial selection criteria	First-generation (cefazolin) or second-generation (cefuroxime) cephalosporins	Third-generation cephalosporins (ceftriaxone) ± nitroimidazoles (metronidazole)	Other antimicrobials (e.g., *β*-lactamase inhibitors, glycopeptides).		
Current standard protocols (or routine choices) for perioperative prophylactic antimicrobials of Class I incisions	97 (67.8%)	27 (18.9%)	19 (13.3%)		
Antimicrobial selection criteria	Yes	No	Uncertain		
Microbiological cultures are performed prior to prescribing antimicrobials if patients present with symptoms of incisional infection	135 (94.4%)	0 (0.0%)	8 (5.6%)		
Multidisciplinary consultations are initiated upon encountering infections with resistant bacteria	130 (90.9%)	5 (3.5%)	3 (2.1%)		
The duration of antimicrobial therapy is extended when encountering resistant bacterial infections	51 (35.7%)	32 (22.4%)	70 (49.0%)		
Availability of dedicated clinical pharmacists participating in perioperative antimicrobial stewardship and providing consultations	139 (86.7%)	1 (0.7%)	3(2.1%)		

Regarding the duration of therapy, fewer than 40.0% of the participants reported the practice of extending perioperative antimicrobial administration beyond recommended protocols. Clinical pharmacy consultations were actively sought by 86.7% of respondents to refine and optimize perioperative antimicrobial regimens.

In summary, while the overall clinical practice of perioperative antimicrobial prophylaxis among pediatric surgeons in Jiangsu Province appears standardized, clinical deviations in drug selection and duration control persist among some practitioners, necessitating further refinement and adherence to guidelines.

### Individual-level KAP association analysis (likert-based scoring)

**Figure 2:**
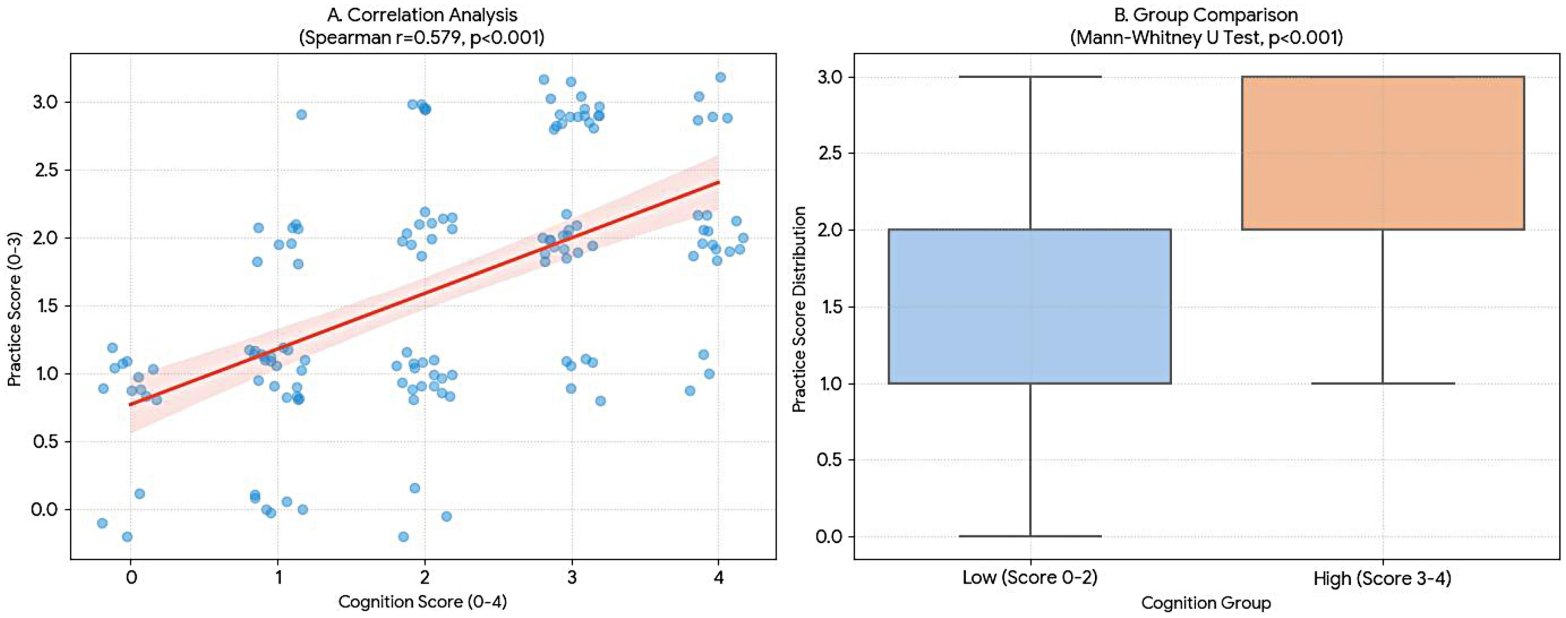
Individual-level KAP association analysis. **(A)** (Correlation Analysis): Illustrates the regression relationship between cognition scores and practice scores among 143 surgeons. The red line represents the regression trendline, and blue dots represent individual physicians. **(B)** (Group Comparison): Displays the differences in practice scores between high-knowledge and low-knowledge groups. The boxplot demonstrates that the median practice score of the high-knowledge group is significantly higher than that of the low-knowledge group.

This study conducted an individual-level association analysis of Knowledge, Attitudes, and Practices (KAP) regarding perioperative antimicrobial prophylaxis among the 143 surveyed surgeons (Figure 2). A quantitative analysis was performed to evaluate the correlation between surgeons' cognitive scores and their clinical practice scores. Spearman's rank correlation analysis revealed a significant positive correlation between the participants' total knowledge scores and their total practice scores (*r* = 0.579, *P* < 0.001). Subgroup comparisons further demonstrated that the mean practice score in the high-knowledge group (Score ≥ 3) was significantly higher than that in the low-knowledge group (2.22 ± 0.8 vs. 1.23 ± 0.9, *P* < 0.001). These findings underscore the fundamental value of medical education in antimicrobial stewardship: in an idealized context devoid of external interference, enhancing clinicians' cognitive understanding of perioperative prophylaxis effectively drives improvements in clinical behavior.

### Statistical analysis of knowledge-practice concordance

**Figure 3:**
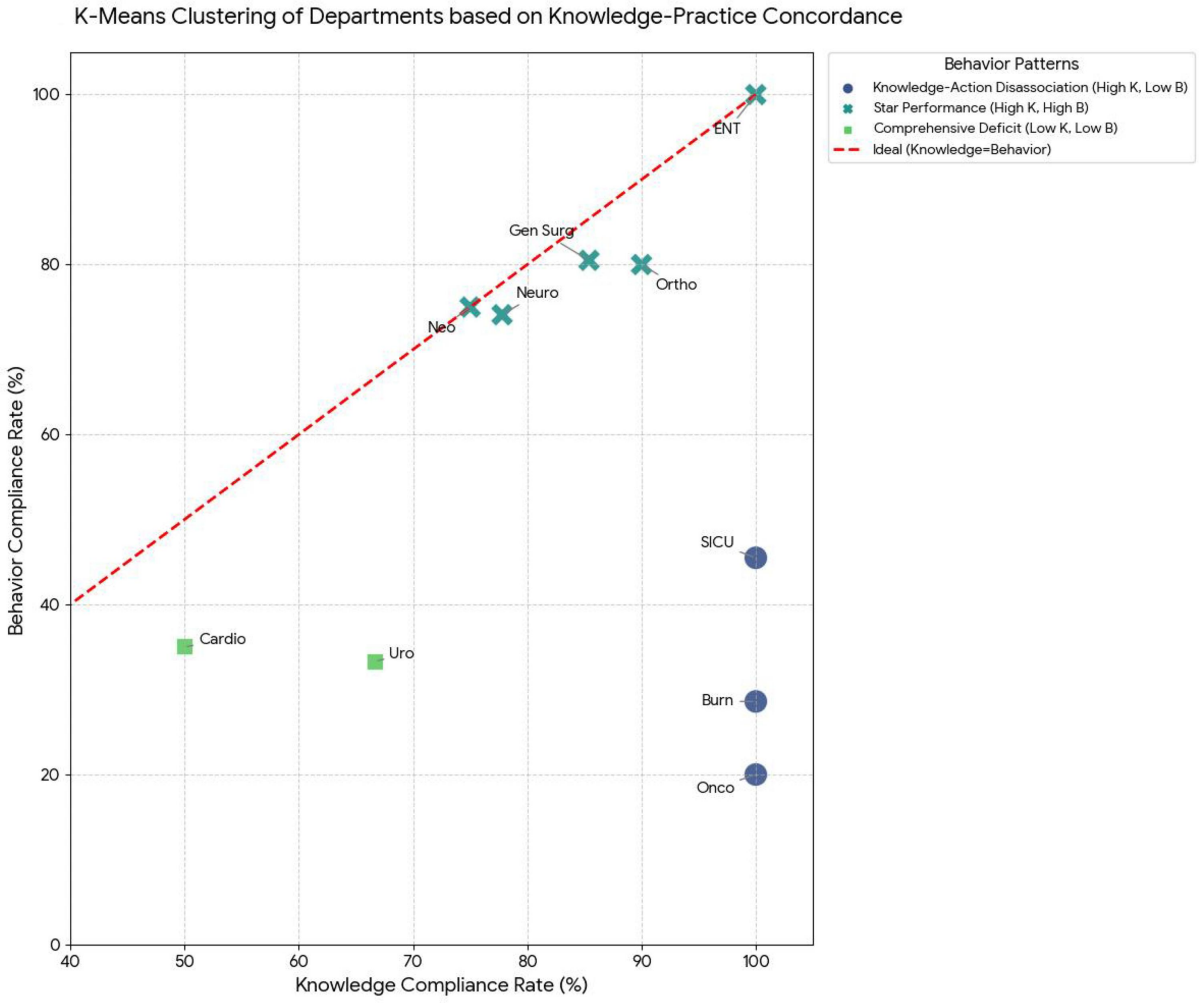
Department-level cluster analysis based on knowledge-practice concordance (K-means clustering).

Across clinical departments, knowledge rates regarding perioperative antimicrobial prophylaxis were significantly higher than practice compliance rates (Wilcoxon signed-rank test, *P* = 0.0117); Pearson correlation analysis revealed no significant linear relationship between knowledge and practice rates at the departmental level (*r* = 0.1214, *P* = 0.7383). Subsequent K-means clustering categorized the 10 clinical departments into three statistically distinct behavioral clusters (Figure 3), characterized as follows: (1) Star Performance Cluster: Characterized by high levels of both knowledge (mean = 88.5%) and practice compliance (mean = 85.8%), with data points closely distributed along the y = x reference line; (2) Knowledge-Practice Disassociation Cluster: Demonstrating a high knowledge rate (100.0%) contrasted by a low practice compliance rate (31.3%), representing a classic “high-knowledge, low-execution” profile. (3) Comprehensive Deficit Cluster: Exhibiting knowledge (58.3%) and practice compliance rates (34.2%) significantly below the institutional average, manifesting as dual deficiencies in both cognitive reserves and clinical execution regarding perioperative antimicrobial prophylaxis.

## Discussion

### Current status of perioperative antimicrobial prophylaxis and bottlenecks in the management of discontinuation timing

Current data indicate that pediatric surgeons in Jiangsu Province possess a high overall cognitive level regarding the rational use of perioperative antimicrobials, with 81.82% of respondents having received systematic specialized training. Nevertheless, at the level of clinical execution, the “timing of discontinuation” for prophylactic medication remains a significant weakness in standardized management. The survey revealed that only 74.8% of physicians were willing to adhere to the protocol of discontinuing medication within 24 h postoperatively for Class I (clean) surgical incisions. These findings demonstrate that surgical Antimicrobial Stewardship (AMS) has transitioned from a broad management phase focused on “whether to medicate” to a refined management phase centered on “when to discontinue” (8). This discrepancy reflects a confusion between “prophylactic” and “therapeutic” medication within clinical practice among some physicians. Some practitioners cite the degree of surgical trauma (e.g., cardiac or spinal surgery) or the use of implants (e.g., in Pierre Robin sequence) as a rationale for extending the duration of prophylaxis. From pharmacokinetic and microbiological perspectives, the core objective of prophylaxis is to maintain adequate tissue drug concentrations during and shortly after surgery to prevent bacterial colonization, rather than to treat latent infections. Guidelines indicated that extending the duration of postoperative prophylaxis fails to provide additional infection control and instead exacerbates the global spread of antimicrobial resistance (6, 9). Consequently, correcting the cognitive misconception that “extended medication enhances safety” is the primary task for the current refined management of perioperative antimicrobials.

### The driving capacity of individual cognitive reserves on clinical practice standards

Quantitative analysis at the individual level demonstrated that surgeons' cognitive level is a central driver of adherence to standardized clinical practices. Spearman correlation analysis demonstrated a significant positive correlation between knowledge and practice scores (*r* = 0.579, *P* < 0.001), suggesting that adequate knowledge is a prerequisite for appropriate clinical decision-making (10, 11). Further group comparisons revealed that mean adherence to clinical practice standards was significantly higher in the high-knowledge group compared to the low-knowledge group (*P* < 0.001). This suggests that certain non-standard practices, such as incorrect timing of administration or neglect of postoperative reassessment, stem from an insufficient mastery of guideline details or gaps in awareness of antimicrobial resistance risks. The high training coverage rate of 81.8% in this study played a critical role in reducing unintentional deviations from best practice attributable to information asymmetry. However, the correlation coefficient (*r* = 0.579) indicates that although improvements in knowledge substantially enhance basic procedural compliance, they account for only part of the variability in clinical behavior. In complex clinical scenarios, surgeons' decisions may also be influenced by non-cognitive factors, including departmental culture and risk-avoidance psychology.

### Analysis of overall behavioral patterns

K-means clustering was used as an exploratory analytical approach in our study. Although no significant correlation was observed between departmental knowledge and practice rates (Pearson *r* = 0.12, *P* > 0.05), the clustering results suggested the presence of several distinct behavioral patterns. Notably, departments such as oncology, burns/plastic surgery, and the surgical intensive care unit (SICU) demonstrated high levels of guideline awareness but relatively low practice adherence, which may be influenced by multiple factors according to previous studies (12–15). However, given the limited number of departments included in the analysis, these findings should be interpreted cautiously and regarded as hypothesis-generating observations rather than definitive classifications. Future studies involving larger samples and additional institutions are needed to determine whether targeted antimicrobial stewardship strategies should be tailored to specific clinical settings.

### Limitations of the study

This study still has several limitations. First, the present study has limitations in sample size, geographic coverage and included departments. With a relatively small total sample of 143 cases and a limited number of participating departments, the stability and reproducibility of the cluster analysis results may be compromised. Further large-sample, multicenter studies covering more institutions and departments are therefore needed to validate our findings and verify the robustness of the identified behavioral patterns. Second, there is potential for self-reporting bias, as the data were derived from questionnaires based on physician self-reports. This may lead to an unconscious social desirability bias, potentially resulting in actual clinical non-compliance rates being higher than those observed in this survey. Third, the analysis focused on process indicators such as knowledge and behavior and did not incorporate clinical outcomes, including infection rates or antimicrobial resistance patterns, which should be addressed in future cohort studies.

## Data Availability

The original contributions presented in the study are included in the article/Supplementary Material, further inquiries can be directed to the corresponding author/s.
